# Triple careers of athletes: exploring the challenges of planning a pregnancy among female elite athletes using semi-structured interviews

**DOI:** 10.1186/s12884-022-04967-7

**Published:** 2022-08-15

**Authors:** Pavel Dietz, Larissa Legat, Matteo C. Sattler, Mireille N. M. van Poppel

**Affiliations:** 1 Institute of Occupational, Social and Environmental Medicine, University Medical Centre, University of Mainz, Mainz, Germany; 2 Institute of Human Movement Science, Sport and Health, University of Graz, Graz, Austria

**Keywords:** Pregnancy, Maternity, Elite athlete, Women, Career

## Abstract

**Background:**

The challenging factors that elite athletes perceive for combining their sportive career with planning a pregnancy and motherhood need to be identified in order to develop supportive measures. Therefore, this phenomenological qualitative study aimed to explore challenges associated with planning a pregnancy among female, non-pregnant elite athletes.

**Methods:**

Semi-structured skype-interviews were performed among female elite athletes (athletes competing on national or international level) aged 28 years or older. Using Mayring’s qualitative content analysis approach, anchor examples served to identify potential challenges of planning a pregnancy which were categorized independently by two researchers.

**Results:**

Interviews of 16 elite athletes (mean age 30.7 years) entered analysis. Eleven challenges of planning a pregnancy were identified, categorized into organizational / environmental, financial, personal, and physical factors.

**Conclusions:**

With regard to financial challenges, we propose mandatory maternity leave and continuation of the contracts and salary. Furthermore, mentoring programs may help to provide support and advice to new generations of female elite athletes and help to reduce concerns regarding the wish of becoming pregnant during a sportive career. In order to reduce physical concerns regarding pregnancy and exercise, we see a need for scientific studies investigating the association of sport discipline specific characteristics on sportive performance and the mother´s, fetus’ and child´s health. Finally, the results of the current study may be used in future quantitative studies to test specific hypotheses.

## Background

Reaching and remaining at the elite level in competitive sport require athletes, and also those around them, to invest at different levels (e.g., physical, social, financial) during a long period of time [1]. Consequently, being an elite athlete is often at the expense of education, work, family, and other interests in life. In this context, a dual career describes the challenge to combine a sporting career with education or work [2]. However, athletes do not only have to face challenges in combining their sportive career and education or work, but increasingly also in combining and planning a family. This balancing act of combining a sportive career, work or education and planning a family can be described as a triple career. Although this issue also concerns male athletes, it represents a particular challenge for female athletes who need to both adjust their training during the pregnancy [3] and postpartum period [4], and manage to return to competitive sport after pregnancy (reconciling motherhood and sport) [5].

It is a goal of the International Olympic Committee (IOC) to promote equal opportunities for girls and women to participate in sports [6]. In recent decades, female participation in competitive and elite sports has increased substantially. This trend is observed at all levels of sports competitions, although with variations between countries and sports [7]. With the growing number of women in competitive sports, including elite sports, and the increasing duration of active sporting careers, there will be a simultaneous increase in the number of athletes who (want to) become pregnant during their sportive career.

Some studies have highlighted pregnancy and motherhood as the main reasons why female athletes may end their sportive career or fail to reach their full potential in sports [8, 9]. Furthermore, the time required for competition and training as well as potential fears and doubts regarding their sportive future, may leave little time for motherhood [10]. Additionally, medical discourses that have positioned exercise and sport training during pregnancy as dangerous may also influence an athlete´s decision of planning a pregnancy [11]. Thus, planning pregnancy may be influenced by factors associated with their sports career. However, these challenging factors are mostly hypothetical as empirical data in elite athletes addressing this issue are lacking. We are aware of only one study performed in a collective of twenty Spanish elite athletes which already had been pregnant. Using a qualitative approach, the authors aimed to make the pregnancy experiences of these athletes visible. In this study, all sportswomen were faced with many fears and doubts concerning both, the present (training and career during pregnancy) and the future (training and career after pregnancy with a baby) [12].

Substantial research on dual careers has been performed in the last years and different approaches (pathways) to realize such a career were developed, for instance the educational/vocational pathway, the parallel dual career pathway or the sporting pathway [13]. In contrast, approaches dealing with triple careers are unfortunately lacking. However, to provide tailored support and allow all female athletes to plan and have a child during their active career, the challenging factors perceived by elite athletes for combining their sportive career with planning a pregnancy and motherhood need to be identified first. In contrast to the above mentioned study [12], we believe it is necessary to investigate attitudes, beliefs and associated challenging factors of female elite athletes (in reproductive age) before having been pregnant. This allows us to identify also the reasons for not planning a pregnancy even though a child-wish may be present (which cannot be identified in female elite athletes who already were pregnant because they may be considered a selective sample regarding their challenging and supporting factors). Therefore, this study aims to explore challenges associated with planning a pregnancy among female, non-pregnant elite athletes.

## Methods

### Study design and interview guide

A qualitative semi-structured interview study was developed by the study team (PD, MvP, MS) with participation of three female student athletes in reproductive age representing the target population. The individual interviews were performed digital using Microsoft skype*®*. Such videoconferencing tools allow researchers to interview geographically dispersed populations (such as an international sample of elite athletes) with a recorded interaction that at least mimics face-to-face interactions [14]. Berg (2009) further stated, that synchronous environments (or videoconferencing in this case), are similar to face-to-face interviews, especially in regards to unstructured or semi-structured interviews [15]. More recently, Janghorban et al. (2014) came to the conclusion, that skype interviewing is the next generation of online synchronous interviewing in qualitative research because of saving resources such as time and costs [16]. The skype-interviews were performed by supervised student researchers who practiced the interview procedure by performing at least two skype-interviews each in advance.

The guide for the semi-structured interviews was structured into the following topics: i) *introduction* (welcome, technical check (sound and camera), short introduction of the interviewer), ii) *description of the study* (study team, eligibility criteria, aim of the study, procedure), iii) *ethical information* (voluntariness and anonymity), iv) *general questions on sociodemographic and sport-related characteristics* (e.g., age, residence, time starting with sport, development of sporting career and greatest achievements, club membership, mean hours of training per week, occupation besides sportive career). After this more general part, the v) *specific questions* addressing the study aim followed. Athletes were first asked if they were ‘in a partnership’ followed by a filter question assessing whether the women ‘currently had the general desire of getting pregnant at some point in time’ (‘yes’ / ‘no’). Then, the interview guide was divided into vi-a) a part for women that currently had the wish of getting pregnant and those vi-b) who had not. Athletes who stated to have the general wish were then asked, ‘if the period of becoming pregnant would be foreseeable’ (‘yes’ / ‘no’). Those who answered with ‘yes’ vii-a) were then asked ‘when this period would be’, ‘why exactly that specific period would be appropriate’, and ‘why the right period has not been appropriate (until now)’. Those who answered ‘no’ were asked vii-b) ‘on what circumstances the right period of getting pregnant would depend on’ and ‘what would you change in your situation (if you could) so that the right period of getting pregnant would be now’. The last part (viii) included more sensitive questions addressing the *harmonization of sportive career and planning a family*: the athletes with the general wish of getting pregnant were asked viii-a) ‘have you ever tried to get pregnant’, ‘do you think your sportive career can be reconciled with a role as mother’ and ‘in your opinion, are there any challenging factors that make the decision for elite athletes to get pregnant difficult’. Those athletes who had no general wish of getting pregnant were only asked the last question (‘challenging factors’). Finally, at the end of the interview all athletes were asked ix) if there is anything else they would like to say/comment on.

Approval to perform the study was obtained by the ethics committee of the University of Graz, Austria (GZ. 39/60/63 ex 2017/18). Patients or the public were not involved in the design, or conduct, or reporting, or dissemination plans of our research.

### Recruitment and analysis

The aim of this phenomenological qualitative study was to describe the lived experience of female elite athletes in reproductive age. We used non-random purposive sampling based on priori formulated eligibility criteria. These were: i) being a female athlete ii) performing on an elite level (at least competing on a national level), iii) being in reproductive age but not younger than 28 years, and iv) speaking German as mother language. We considered 28 years as relevant age cut-off since the average age of becoming pregnant is 29 years in Germany [17] and 31 years in Austria [18]. Moreover, athletes of that age may have both a better understanding/knowledge of their sporting field and likely already had more concrete thoughts on planning a pregnancy. In addition, we tried to include participants from different sports disciplines to increase variation. The overall aim was therefore to include participants who are able to provide rich information relevant to the research question. As from experience, elite athletes are not easy to recruit as they are a very specific collective having a strict routine, participants were recruited via the broad personal network of the study team and colleagues from other sport faculties, sport organizations, and sport clubs. Potential candidates were contacted with a standardized Email or via telephone in which they were informed about the aim and procedure of the study and asked if they would be available for an interview.

Data analysis was performed by two researchers (LL and PD) using Mayring’s qualitative content analysis approach [19]. According to this approach, the analysis of texts with regard to the respective research questions, in our case exploring challenges of planning a pregnancy among female elite athletes, can be performed deductive or inductive. A deductive approach is used if a research question is built on a strong theory basis and potential categories can already derived from the literature. An indicative approach enables researchers to work on more explorative, theory-building research questions. It is used when potential categories do not already exist and have to be formulated during the analysis. Since our research question was explorative and the topic rarely addressed by previous research, we decided to use the inductive (category-building) approach. The analysis contained the following steps: i) recording of all interviews and transcribing them verbatim, ii) reading all transcripts to get a general sense of all interviews, iii) re-reading the transcripts focusing on the research question by highlighting all text passages that can be interpreted as a potential challenge of planning a pregnancy among female elite athletes. These passages were then iv) extracted and coded by two researchers (LL & PD). To enhance the trustworthiness of study results, bracketing was incorporated prior to and throughout the study, including preparation of the interviewers and pre-testing the interview situation to reduce bias, limiting the literature review and using open-ended questions in the interview guide. Each of the researchers independently coded all the interviews. Codes were compared and reconciliation of discrepant codes was achieved. Anchor examples were extracted for each potential challenging factor of planning a pregnancy. These challenging factors were then v) grouped into categories, independently by two researchers (LL & PD). Discrepancies were resolved by consensus among the two researchers. No qualitative software was used to analyze these data. For presentation in the present paper, anchor examples were translated word-by-word from German into English language.

## Results

Seventeen female athletes fulfilled eligibility criteria and were interviewed. One woman stated at the beginning of the interview that she currently was pregnant and was excluded from analysis. Consequently, interviews of *n* = 16 female elite athletes entered analysis. The women were between 28 and 37 years old (mean age: 30.7 ± 2.4 years), 14 were from Austria and two from Germany. For reasons of anonymity, we did not report the highest sportive achievement in relation to the respective discipline, because this may enable readers to identify single participants. Highest sportive achievement ranged from world champion (*n* = 1), participation in Olympic Games, world or European championships (*n* = 5), national champion (*n* = 2), and participation in national championships (*n* = 8). The disciplines in which they performed were endurance sports (*n* = 6), ball and backstroke sports (*n* = 5), athletics (*n* = 3), dancing (*n* = 1), and crossfit (*n* = 1). Nine participants (56.3%) stated to have the general wish to become pregnant. In total, eleven challenging factors of planning a pregnancy were identified, categorized into organizational / environmental, financial, personal, and physical factors.

### Organizational / environmental

*Environmental support* was named by most of the women as a main condition to successfully combine sportive career with planning or having a family. “I think sport and being mother is only possible if you have a super environment […], otherwise not” (A3: 34 years old, general wish of becoming pregnant) or “[…] really there are exceptions who can handle this, but you need an appropriate environment, people, who help you” (A15: 28 years old, no general wish of becoming pregnant). Other women stated: “[…] you are in the south of the town and have a lactate test, if you don´t have a grandma or whoever, where do you bring your child?” (A12: 37 years old, general wish of becoming pregnant), or “I know some who can handle this, who take the children along with them, with the grandparents” (A17: 31 years old, no general wish of becoming pregnant). These quotes demonstrate that a good environment often means that the athletes have people around that support with child care. And most suitable, these people should not be strangers but come from the personal (family) environment, as further stated by several athletes.

*Time (management)* is another factor named by the athletes as shown for example by the following quotes: “Yes, well in any case lack of time or rather priorities, because I think as an elite athlete you set your priorities often different than normal people. […] and there is time a big factor” (A11: 30 years old, general wish of becoming pregnant). Or “with a child you need a time management, especially later when the child is older, then everything must be planned” (A2: 30 years old, no general wish of getting pregnant). These quotes demonstrate that the factor ‘time’ might be related to the above-mentioned factor environmental support, because a good environmental support may save time.

Quotes such as “especially if you are travelling very much, well, I think this in not for everyone, and you don´t know, if people in this situation think, that they are in a situation where they can have a child at all” (A1: 31 years old, general wish of becoming pregnant) or “because of all the travelling, well, I mean the races are in winter and we are on the road all over the world, and yes, I think that (pregnancy) simply doesn’t fit” (A14: 28 years old, general wish of becoming pregnant) highlight *travelling*, and in this context being away from home, as being another (sport-) organizational barrier. Another woman (A15) said: “First of all […], it is really hard, because you are constantly on the move. My last relationship ended specifically because of that reason”.

### Financial

Although most of the interviewed athletes competed on a high level, *financial insecurity* was stated by most of them. For example, one athlete (A1) stated: “In athletics for example, you earn money at competitions. And if you don´t run, you don´t get money.” Others stated for example, “if you don´t earn money with it (sport), then the combination (sport and motherhood) is difficult” (A3), or “you just cannot live from your sport” (A6). In this context, some athletes identified being employed as an athlete, for example as a sport-soldier or a sport-police woman, makes it much easier: “Federal army is a nice thing, because it is the only opportunity, to finance yourself” (A4: 30 years old, general wish of becoming pregnant). A woman that not had the general wish of becoming pregnant (A7: 28 years old) found really hard words: “If you perform on an elite level, you are in a financial dependency, […] then you make the decision against a child or you push it in the future. Because you are financially depending on sport. That is a big reason”.

Strongly related to financial insecurity is the aspect of *insecurity in sponsoring and contracts*. There might be a large knowledge gap on the side of the athletes what happens with sponsoring and contracts when becoming pregnant: “I know from contracts, where it is stated that you do only receive the whole money, if you perform for example ten competitions in a year. Consequently (if you would be pregnant), they would have an argument not to give you the whole money” (A1), or “what happens if I get pregnant as an elite athlete? For me this is an unresolved question, I mean, if I say my contract is over, […] because it could be that you get pregnant during having a contract, and I don´t know what happens then, and I think it would be delicate to ask” (A4). Others said that it is different between different sponsors as demonstrated by the quote “well, this is hard to answer, because this is very individual if sponsors stand for it (pregnancy) or not” (A14). And again, being in the military was stated as being “good, because it will be supported, being a female athlete in the military” (A12). Overall, this financial insecurity seems to influence female elite athletes in their decision of planning to become pregnant.

### Personal

*Reaching the set goals*, very well demonstrated by the quote “now quickly to the Olympics, then I can get pregnant” (A14) was mentioned by several athletes. Others stated: “As an elite athlete you are already somewhat ambitious and have certain goals, and I think, it is difficult to achieve your goals with a child” (A6) or “I really want to have the feeling to finish my career with a good conscience before entering a new stage of life” (A7).

This overlaps partly with another factor, namely *waiting for the right time*. Beside several others, A4 stated that “well, when I become a mother, then I am mother, and I want to maintain the option, when the right time comes”.

*Finding* and also *having the right partner* was another personal aspect named by several women. Some women stated that it is really difficult to find a partner at all during the sportive career: “Well, a big reason is not having a partner […]” (A8: 31 years old, no general wish of becoming pregnant). Another woman said “well, if you have a partner who is really flexible, has a good job and earns good money, well […], but when your partner is an athlete as well, it would not be possible” (A5).

Some women, especially those who had the general wish of becoming pregnant, were *unsure if they could 100% live up to the mother role*: “I prefer to do what I do with all my heart, so I am from all my heart an athlete, can give everything, and then, when I am a mother, I can give everything for my children” (A1), or “[…] and on the other side, I think as a mother, you want to be there for your child, all the time” (A6).

Quotes such as “that is my opinion, yes. You have to look on nobody, you have no consideration for someone, you can train as long as you want. With a child it could get complicated, it will be complicated” (A12), demonstrate that *keeping autonomy* is also an issue, especially in women not having the general wish of becoming pregnant: “Maybe a kind of loving my freedom, […], and actually, I really love my life as it is, also the sportive part, and I don´t want to combine this, yes” (A5) or “because I am really egoistic and I like my life as it is” (A9: 33 years old, no general wish of becoming pregnant).

### Physical

Several women had concerns regarding their *physical performance* and *health* in combination with a potential pregnancy: “I think if you do elite sports as a mother, there will always come others that surpass the limits and the own performance will decrease and you cannot longer reach the limits, that you reached then (earlier before pregnancy)” (A16: 28 years old, general wish of becoming pregnant). One woman expressed really concrete concerns. She stated that “on a physical level, that a woman after pregnancy comes back is really difficult. The physical performance will decrease, also 2–3 years after pregnancy because of the double function (as mother and athlete)”. Some women said that “being pregnant is the same than being injured”.

In contrast, some women had friends or knew athletes who had really good experiences with pregnancy and returning to physical performance.

## Discussion

Using a qualitative approach, we aimed to identify potential challenging factors of planning a pregnancy among female elite athletes. Eleven potential challenges in four categories were observed. We are going to discuss these challenges in view of their potential of getting changed and provide recommendations and, if available, examples from sports practice for reducing those challenges.

Two barriers which might be strongly related to each other are *financial insecurity* and *insecurity in sponsoring in contracts* resulting in a financial dependency on sport. This supports the findings of Martínez-Pascual et al. (2017) among female elite athletes and Benzies et al. (2006) among women in general who identified socio-economic status and financial stability being relevant motivational factors of women to plan a pregnancy [12, 20]. One approach to reduce this financial pressure on athletes is the model of ‘professional athletes’ for the military or the police, a common model for example in Germany and Austria. For example, as part of the ‘Spitzensportförderung’ of the *German Federal Armed Forces* (Deutsche Bundeswehr) in collaboration with the *German Olympic Committee* (Deutscher Olympischer Sportbund, DOSB), 744 athletes are funded with more than 30 Million Euro annually as so called sport soldiers for participation in Olympic sports. This model provides financial security for the athletes as they receive a fixed salary and have a secure job. However, of the 744 funded athletes in 2018, only 243 (32.7%) were women [21]. As federal funding for athletes is always limited, we see a large responsibility for the sports organizations. One positive example was the *Federal International Football Association* (FIFA) who introduced two landmark reforms to strengthen the protection of female football players and coaches in 2020. With regard to the players, this includes a mandatory maternity leave of at least 14 weeks at a minimum of two thirds of the player’s contracted salary. Furthermore, football clubs can assign temporary replacements but must reintegrate players when they return from maternity leave and provide adequate medical and physical support [22]. A comparable approach was introduced by the *Women's National Basketball Association* (WNBA) in the United States announcing a new collective-bargaining agreement with its players’ association aiming to increase salaries significantly and guarantee players fully paid maternity leave for the first time in its history [23]. However, with the exception of these prominent examples of large sports organizations, paid maternity leave and a guaranteed reintegration afterwards are not common in professional sports. Therefore, we argue for general standards in professional sports regulating maternity leave and salary of pregnant elite athletes.

In addition, several women had health concerns when training and competing during a potential pregnancy. A fundamental problem is the lack of evidence, the lack of consensus and the presence of more or less manifest myths regarding exercise, especially strenuous exercise, in pregnancy [24]. For example, concerns about the fetus, preeclampsia, gestational diabetes mellitus, preterm delivery, gestational weight gain, birth weight, and injuries for pregnant elite athletes exercising strenuous are reported in the literature [25]. Consequently, athletes as well as their trainers often do not know how intense and how long they could perform their training in pregnancy. Health care providers are sometimes overcautious and recommend reducing exercise intensity and/or duration [25]. A systematic review and meta-analysis including 135 studies concluded that on the one hand, prenatal exercise is safe and beneficial for the fetus. And on the other hand, maternal exercise is associated with reduced odds of macrosomia (abnormally large babies) and not associated with increased odds of neonatal complications or adverse childhood outcomes [26]. This is also reflected in the recommendations of the American College of Obstetricians and Gynecologists (ACOG) [27], who recommend 30 min of moderate physical activity per day for women with an uncomplicated pregnancy. However, these recommendations are formulated for women of the general population and not for the specific collective of elite athletes. Another recent systematic review and meta-analysis exclusively addressing the effects of vigorous intensity exercise in the third trimester of pregnancy came to a similar conclusion [24], namely that vigorous intensity exercise in the third trimester appeared to be safe for most healthy pregnancies. However, studies among elite athletes are lacking, and therefore it is not possible to formulate recommendations for this group, yet. Since different sportive disciplines can rarely be compared, we recommend further research addressing the effects of the specific training characteristics of different sport disciplines (e.g. type, intensity, duration) in pregnancy on pregnancy outcomes. For the moment, we support the recommendations of Pivarnik et al. (2016) that elite athletes who wish to continue strenuous exercise during pregnancy should have a clear understanding of what it means to exercise during pregnancy, should be able to recognize feedback from their body and should have approval from their health care providers [25].

Under the *organizational / environmental category* the challenging factors “environmental support”, “time management” and “travelling” are summarized. These factors are highly associated with each other and the factor “time” can be seen as overreaching. For instance, if one does not have an environment that supports you by caring for your child, as stated by some women, you do not have the time for training and competitions. The same may apply for travelling and having poor time management, both factors may reduce the time for child caring. With regard to these factors, a supportive environment can have a buffering role. The described challenges in this study are well-known from studies exploring the association between motherhood and career progression in other occupational settings where the factors time as well as age and number of children were highlighted as significant factors limiting women’s career progress [28]. Whether athletes have a supportive environment is difficult to modify by organizational changes. One approach could be to provide advice for elite athletes and their environment how to manage and to deal with the double role as a mother and a successful athlete, as also recommended for example for mothers in academic careers [29]. Furthermore, sports organizations and those organizing large events (e.g. Olympics, Wimbledon) could be more aware of the needs that women with children have and could provide them a supportive environment during the events, for example by organizing day care opportunities or nannies.

Under the *personal category*, the factors ‘reaching the set goals’, ‘waiting for the right time’, ‘having the right partner’, and ‘being unsure if they could 100% live up to the mother role’ are summarized, whereas ‘having the right partner’ may also be strongly related to the ‘supportive environment’ in the previous category if the partner helps in child caring. However, not having a partner may also be a reason for not having a child or the general wish of planning a pregnancy. A survey study addressing pregnancy and motherhood during surgical training, which included 347 female surgeons with 452 pregnancies identified similar concerns as described by our participants such as difficulties in balancing pregnancy and motherhood with surgical training. An interesting finding in that study was that breastfeeding was important to 95.6% of the surveyed women, yet 58.1% stopped breastfeeding sooner than they wished [30]. This point might be related to uncertainties regarding the mother role, as revealed by our study. With the aim of attracting and retaining the most talented candidates for surgical programs, the same is intended in elite sports, they argued that the challenges facing new mothers in surgical residency must be addressed and recommend mentoring programs for female medical students. Such mentoring programs may also be helpful to face challenges and concerns regarding pregnancy in elite athletes. For example, former elite athletes who successfully managed the balancing act between motherhood and having a successful career could give their positive and negative experiences to the next generation of female elite athletes. Furthermore, they could support sports organizations and stakeholders in creating the conditions for elite athletes as positive as possible.

Although we want to avoid any kind of quantitative interpretation of our qualitative results, it has to be stated that athletes with the general wish of becoming pregnant reported more barriers of becoming pregnant compared to those who did not have this wish. In our opinion this seems plausible because women with a wish to become pregnant may think more about this topic and how they would deal with a potential pregnancy or a child compared to those who do not have this wish. However, we feel that including the athletes who did not want to become pregnant is a strength of our study, since it is relevant to know the factors on which they based their decision, and whether it is related to their status as elite athlete. All previous studies only included athletes who had already made the decision to start a family, and, thus, could not address this issue. As a potential limitation of the present study, it has to be mentioned that the 16 qualitative interviews were performed in a very specific sample namely female elite athletes from Germany and Austria. Therefore, the assumptions of the present study should be verified by future quantitative studies that test specific hypotheses among a geographically more diverse sample of female elite athletes.

## Conclusions and recommendations

Using a qualitative approach, we assessed challenging factors of planning a pregnancy among female elite athletes and provide recommendations for reducing those challenges. With regard to the financial challenges, we see a strong responsibility for the sports organizations and sponsors. Mandatory maternity leave, continuation of the contracts and salary as well as the model of ‘professional athletes’ for the military or the police may help to reduce the finance-related concerns on getting pregnant. With regard to the physical concerns, we argue for a wide range of scientific studies investigating the association of discipline specific characteristics on sportive performance and the mother´s, fetuses and child´s health. With regard to the environmental / organizational and personal challenges, mentoring programs may help to provide support and advice to new generations of female elite athletes and help to reduce concerns regarding the desire of becoming pregnant and having a sportive career.

## Data Availability

According to the responsible ethical committee (see above), the University of Graz is the data holding organization. The institution is not allowed to share the data publically in order to guarantee anonymity to the very specific collective of female elite athletes in the present qualitative study because some sport- and competition-specific information could be linked to specific participants (for example: World Champion in snowboarding in 2018). The transcripts of the present qualitative study (written and audio) are stored on the institution server at the University of Graz and can be requested for scientific purposes via the institution office (bewegungswissenschaften@uni-graz.at) or the corresponding author of this study (pdietz@uni-mainz.de).

## Abbreviations

ACOG: American College of Obstetricians and Gynecologists
DOSB: Deutscher Olympischer Sportbund
FIFA: Federal International Football Association
IOC: International Olympic Committee
WNBA: Women’s National Basketball Association
